# CAPformer: Pedestrian Crossing Action Prediction Using Transformer

**DOI:** 10.3390/s21175694

**Published:** 2021-08-24

**Authors:** Javier Lorenzo, Ignacio Parra Alonso, Rubén Izquierdo, Augusto Luis Ballardini, Álvaro Hernández Saz, David Fernández Llorca, Miguel Ángel Sotelo

**Affiliations:** 1 INVETT Research Group, Universidad de Alcalá, Campus Universitario, Ctra, Madrid-Barcelona km, 33, 600, 28805 Alcalá de Henares, Spain; ignacio.parra@uah.es (I.P.A.); ruben.izquierdo@uah.es (R.I.); augusto.ballardini@uah.es (A.L.B.); alvaro.hernandezsaz@uah.es (Á.H.S.); david.fernandez-llorca@ec.europa.eu (D.F.L.); miguel.sotelo@uah.es (M.Á.S.); 2 Joint Research Center, European Commission, 41092 Seville, Spain; 3 INVETT Research Group, Colegio de San Ildefonso, Universidad de Alcalá, Plaza de San Diego s/n, 28801 Alcalá de Henares, Spain

**Keywords:** pedestrian, prediction, action classification, transformer, deep learning, autonomous vehicles

## Abstract

Anticipating pedestrian crossing behavior in urban scenarios is a challenging task for autonomous vehicles. Early this year, a benchmark comprising JAAD and PIE datasets have been released. In the benchmark, several state-of-the-art methods have been ranked. However, most of the ranked temporal models rely on recurrent architectures. In our case, we propose, as far as we are concerned, the first self-attention alternative, based on transformer architecture, which has had enormous success in natural language processing (NLP) and recently in computer vision. Our architecture is composed of various branches which fuse video and kinematic data. The video branch is based on two possible architectures: RubiksNet and TimeSformer. The kinematic branch is based on different configurations of transformer encoder. Several experiments have been performed mainly focusing on pre-processing input data, highlighting problems with two kinematic data sources: pose keypoints and ego-vehicle speed. Our proposed model results are comparable to PCPA, the best performing model in the benchmark reaching an F1 Score of nearly 0.78 against 0.77. Furthermore, by using only bounding box coordinates and image data, our model surpasses PCPA by a larger margin (F1=0.75 vs. F1=0.72). Our model has proven to be a valid alternative to recurrent architectures, providing advantages such as parallelization and whole sequence processing, learning relationships between samples not possible with recurrent architectures.

## 1. Introduction

### 1.1. Context

Road safety is one of the main concerns in the world, being the eighth leading cause of death and the first among young people between 5 and 29 years old. Traffic accidents caused approximately 1.35 million deaths and between 20 and 50 million of non-fatal injuries worldwide in 2016. The social and psychological problems arising from road accidents are also followed by a considerable impact on the economy, costing 3% of gross domestic product in most countries [1].

Vulnerable road users (VRUs) represent more than half of all these deaths. Pedestrian Vulnerable Road User (VRU) group is the most affected one in urban roads in European Union, with 40% of the total VRUs deaths, as stated by European Transport Safety Council (ETSC) [2].

Thanks to various European Union (EU) initiatives and actions, the number of road fatalities has been decreasing since 2011 following a promising and continuous trend. However, it has not been enough to achieve the initial target of 50% reduction by 2020. To continue this reduction, a new ambitious long-term goal has been established, pursuing zero road deaths by 2050. The main tools proposed to reach this goal, try to accommodate human error: highlighting better legislation around risks, such as speeding, drinking while driving, and mobile phone distractions. In addition to these measures, automated, and Autonomous Vehicles (AVs) can provide a reliable platform to help increase human safety within the traffic scenario.

### 1.2. Motivation

Car-to-pedestrian impacts are one of the most frequent accidents occurring in urban environments due to the high level and intensity of interactions between vehicles and pedestrians. Car manufacturers are developing Autonomous Emergency Braking (AEB) systems [3] to support the driver in avoiding when possible (by autonomous braking) or mitigating such crashes. These systems, which depend on the accuracy of the pedestrian detection system, can be much more effective if they can anticipate the intention or action of pedestrians to cross, as an experienced driver does.

Understanding human behavior has been widely studied through trajectory prediction systems (see [4] for a detailed overview). However, as discussed in [5], only using kinematics and positions of humans is not enough to anticipate human action, since global awareness of the scene is necessary, through semantic 3D maps. Although trajectory-based are suitable for this task, they need annotated 3D data and map information. To the best of our knowledge, there is currently no public dataset with these characteristics. As an alternative, camera sensor data can be used for behavior understanding at a higher level, treating the problem as a classification task and establishing as optional the use of maps.

The understanding of human behavior from video data, also known as video action recognition, has experienced considerable development over the last decade due to the rise in popularity of deep learning and the lower cost of hardware (read [6] for a more detailed overview). These methods rely on hidden patterns from camera sensor data to predict the correct action being performed by humans, exploiting both temporal and spatial data features.

Recurrent models have been one of the main options in temporal tasks over the last years. However, since the proposal of the Transformer architecture [7], this new architecture has increased in popularity due to its major improvements in language translation and other natural language processing tasks. These models benefit from parallelization and input sequences are processed as a whole, reducing the risk of forgetting past information.

Recently, the first pedestrian benchmark [8] for crossing action prediction has been released. This work helps in the standardization of the results of the proposed models in the task, allowing a just comparison by using the same splits of data sequences and the same processing techniques.

In this work, we propose a new multi-branch model where all non-image information is processed by a transformer-based model. As far as we are concerned, this is the first method that proposes a model based on this architecture. Although our proposed model follows a similar pipeline to the best performing models in crossing action prediction problem, we innovate in the blocks rather than in creating a different pipeline, including novel methods for both video and kinematics branches. In the video branch, we confirmed that TimeSformer model, based on transformer architecture, is suitable for encoding video information and it is successful in a fine-tuning strategy and that 3D-shifts modules proposed in RubiksNet network can considerably minimize the size of the model with respect to 3D convolutions, maintaining comparable results. In the kinematics branch, the usage of transformer encoder architecture accelerates training with parallelization and allows the computing of relationship among all samples in input sequence. In addition, instead of using a different encoder for each feature, we used a single encoder which combines all of the kinematic features. In our proposed model, we use the previous benchmark for training and evaluation, but we propose alternative pre-processing techniques and strategies for the different sources of input data, to increase the performance before the optimization of the model. We perform a set of experiments to quantify the effect of these pre-processing techniques and empirically highlight problems found with some of the input features. Finally, to validate the proposed architecture, we compare it with the one proposed in the benchmark, reaching state-of-the-art results.

## 2. Related Work

Previous work on the pedestrian crossing action prediction task has been structured by dividing it into subsections. In each subsection, a dataset is introduced along with the methods that use it in their experiments.

### 2.1. JAAD

In the field of AVs, several datasets contain pedestrian annotations that allow research on pedestrian detection and tracking, which leads to trajectory prediction. However, behavior information is not a common feature in these datasets. One of the first publicly available dataset with behavior annotations was JAAD [9] (details in Table 1). For this dataset, the authors proposed a group of baseline models based on convolutional neural networks [10] focused on learning context and behavioral cues from a pedestrian to use it to classify its crossing behavior. However, this approach is based solely on the current frame without temporal information. From this work, most of the proposed systems rely on temporal information, showing an improvement in the results.

In [11], pedestrian 2D pose keypoints sequence from a specific time interval (≈0.5 s) is used as an input to classify, with a random forest model, the pedestrian crossing action being performed. In [12], precomputed features extracted from the complete sequence of pose keypoints are used as input for a multi-branch 2D CNN network. In [13], whole sequences of pedestrian’s keypoints are used in a similar way to the previous one, but generating adjacency matrix representations based on the pose graph, as an approximation to graph learning. Although the previous pose-based approaches rely on temporal data, they do not use temporal models.

Most of the recent methods on the topic use temporal models. Among them, we can highlight two commonly used architectures: recurrent neural networks and 3D CNNs.

One pure example of RNNs is [14], where an LSTM-based encoder-decoder architecture is used to forecast future pedestrian bounding box coordinates and binary intention at each future timestep. In [15], a combination of 2D CNN for image feature extraction (using pre-trained models) and bidirectional RNNs (LSTMs and GRUs) are used for crossing action prediction in a fixed future horizon (1s) using a fixed input temporal window of ≈0.5 s. Ghori et al. [16] also combine a 2D CNN model with an LSTM network, but using the CNN for extracting pose information. Another example of this combination of 2D CNN and RNN is explored in [17], where a multi-task model is proposed for action classification, final crossing action, and the future bounding box coordinates forecasting. Reference [18] proposes another multi-task approach based on this combination, where current action recognition is predicted along with time to cross regression.

In [19], a 3D CNN is used as a classifier at the end of a pipeline for pedestrian crossing behavior, which includes detection and tracking. The 3D convolutional model is trained with the cropped pedestrians’ bounding boxes detected. On [20], a pedestrian crossing intention recognition (PCIR) framework is proposed, which jointly infers both pedestrian crossing intention and scene perception. Scene perception is performed using an object detector, which filters target pedestrians by only attending to pedestrians standing on curbsides. Target pedestrian crossing intention is inferred using a 3D CNN. In addition to the previous modules, distance information extracted from image data is encoded and fused with the feature vector obtained with the previous 3D CNN. In [21] an end-to-end method based on the previous one is developed improving its results by including pose information. 3D CNNs are also used for video frame prediction as a previous step towards crossing classification [22]. In [23], 3D networks are combined with convolutional LSTM layers. However, these methods train the action classifier with whole future frames, treating equally all the pedestrians on the scene.

### 2.2. TITAN and STIP

Although the previous work evaluated their models in JAAD, there are other datasets with behavioral data. TITAN [24], is a relatively large dataset recorded entirely on the streets of Tokyo. It contains fine-grained labeling of road agents’ actions. In [24] they also propose a model which combines 2D and 3D CNNs and RNN models, however crossing action is not the task in this approach.

Another dataset with crossing behavior, binary annotated in this case, is STIP dataset [25]. This dataset is recorded with a multi-camera setup and includes hand-labeled bounding boxes at a low annotation rate. A tracking algorithm is used for sequence creation and to interpolate the annotations at a higher rate. Reference [25] proposes a graph-based approach where a scene graph is created for each pedestrian sequence to benefit from environment information. After graph creation, a GRU recurrent model is used to anticipate crossing action. Although these datasets are a good alternative to JAAD, they have restrictive terms of use.

### 2.3. PIE

In [26], the creators of JAAD released a new dataset, called PIE (details in Table 1), recorded continuously in a single day in Toronto, with clear weather. In addition to label more relevant objects than in JAAD, it includes ego-motion information of the car. This new information is used in combination with estimated pose keypoints, bounding box coordinates, and bounding boxes image crops to train a stacked GRU model in [5]. In the same release article of PIE [26], a multi-task and multi-branch model is proposed. It combines the use of LSTM layers for non-image information (bounding box coordinates and ego-motion information through ego-vehicle OBD measures) and convolutional LSTM layers for image information (bounding boxes crops). In addition to the intention estimation, which uses human responses statistics as ground truth, the model predicts future bounding box coordinates and ego-vehicle speed.

### 2.4. PePScenes

Due to the lack of 3D information in all the previous datasets, PePScenes [27] extends nuScenes dataset [28] with behavioral annotations. A multi-branch model is proposed in the same work which uses 3D trajectory, ego-motion, full-frame image sequences, and map information as input data to a combination of 2D CNNs and LSTMs. A graph-based model is proposed in [29]. It uses PePScenes’ 3D information as input to model pedestrians’ interactions with their environment through clustering and importance weighting.

### 2.5. Benchmark

Machine learning approaches discussed before, mainly based on deep learning, show an increasing trend of crossing behavior understanding tasks. However, the lack of standardization in the evaluation of the models makes it virtually impossible to compare all of them. Kotseruba et al. address this problem with the creation of a benchmark based on JAAD and PIE datasets [8]. Several baselines are evaluated in it and a newly proposed model called PCPA, combining a 3D CNN for video sequence embedding and GRU layers for pose keypoints, bounding box coordinates, and ego-vehicle speed. The attention mechanism is also included in the model as an enhanced feature fusion strategy. In [30], the authors use a model based on PCPA which includes semantic information among the input features. However, they only performed the evaluation on the JAAD dataset and without including semantic information in the PCPA model for a fair comparison.

## 3. Proposed Approach

In this work, we propose CAPformer, a novel multi-branch deep learning temporal model based on transformer architecture rather than recurrent layers. By avoiding recursion, our transformer encoder processes input sequences as a whole, learning all possible intersample relationships, and leaving no room for a possible loss in information.

### 3.1. Problem Formulation

Pedestrian crossing behavior anticipation is a special case of action recognition where the model, instead of predicting the action performed through the video, predicts the final action of the sequence with a limited observation interval as input.

This problem belongs to the group of binary classification, where the final pedestrian crossing action at TTE=0 (time to event) is inferred. As input features, a fixed-length sequence of *N* frames is used. Sequences are extracted from the end part of pedestrian’s tracks, being the last frame of each sequence ($f_{N-1}$) between 1 and 2 s before the event. In the case of the data used, all video sequences are recorded at a framerate of 30 fps. Therefore $f_{N-1}\in \left[\mathrm{TTE}-60,\mathrm{TTE}-30\right]$). The output of each sequence is a probability for each class, crossing and not crossing which sum 1.

### 3.2. System Description

**Figure 1:**
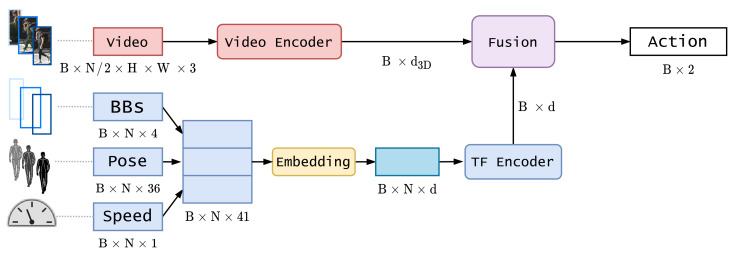
Detailed model diagram. B stands for batch size, N is the sequence length, H is image height, W is image width, BBs is bounding boxes coordinates.

Our proposed model is composed of three main blocks: two encoding blocks and one fusion block. Figure 1 illustrates a detailed diagram.

#### 3.2.1. Video Encoder Branch

The objective of the video encoder is to obtain an embedding vector of size $d_{3D}$ for each video sequence in the batch. We have explored two architectures for this task: RubiksNet [31] and TimeSformer [32]. All of them use sequences of length N/2 (N=16 in the benchmark and our experiments). This length is the one used for training the pre-trained models.

RubiksNet is a network capable of obtaining state-of-the-art results while maintaining a high processing rate, thanks to its reduced number of parameters and operations. This is achieved by using a learnable 3D spatiotemporal shift operation. On the other side, TimeSformer is a slower network based on transformer architecture, which has been introduced in the computer vision field and has achieved state-of-the-art results comparable or even better than convolutional approaches (see [33] for a more detailed classification of transformer architectures in computer vision field). The main reason for using TimeSformer is the need for a reproducible environment for experimentation since RubiksNet contains custom CUDA blocks that use non-deterministic operations. We selected TimeSformer as the reproducible solution to check if a fully transformer-based architecture can achieve comparable results with the ones achieved by the current state-of-the-art method PCPA [8].

#### 3.2.2. Kinematics Encoder Branch

This branch is in charge of encoding a heterogeneous group of features composed by 2D bounding box image coordinates, extracted directly from ground truth annotations, 2D image coordinates of pose keypoints, obtained as the output of a pose estimation algorithm, and ego-vehicle speed, a continuous variable obtained from the ground truth annotations extracted from an OBD sensor. In Figure 1, the last dimension of each kinematic feature represents the raw dimension of each sample in the sequence. In the case of bounding boxes (BBs) coordinates this number depends on the strategy used (see Section 4.1.2). For pose information, 36 value corresponds to 18 pairs of 2D coordinates, representing the image localization of the 18 output keypoints of the pose estimation model. Since the three features have different ranges, we normalized them using min–max scaling for bounding boxes and pose keypoints, where the minimum and maximum values are obtained from the image dimensions (maximum 1920 for x coordinate and 1080 for y coordinate and minimum 0 for both coordinates), obtaining values between 0 and 1. Ego-vehicle speed is *z*-score normalized (or standardized) by subtracting the mean and dividing by the standard deviation of the training set.

**Figure 2:**
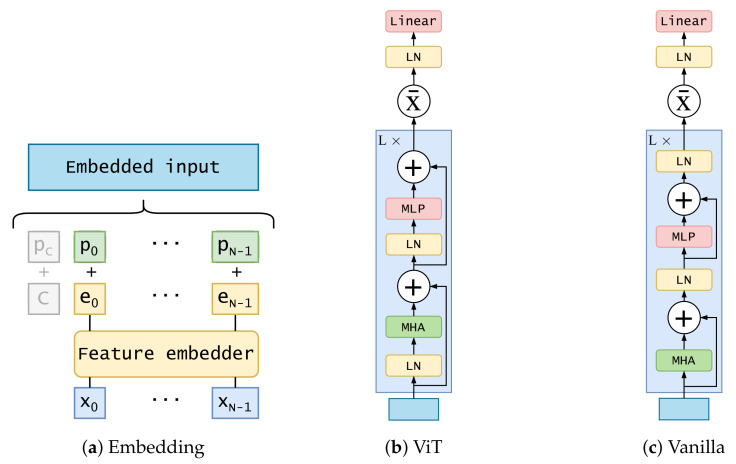
Detailed diagrams of embedding procedure and transformer encoder architectures. MHA stands for multi-head attention, LN for layer normalization, MLP for multi-layer perceptron. Grey part of embedding diagram corresponds to the class token, which is used in ViT encoder.

This branch is based on different variants of the transformer encoder architecture proposed initially in [7]. Two different encoder architectures (composed of *L* layers) are proposed:

- **ViT encoder**: similar to the one proposed in [34] for image classification. It applies a layer normalization on the embedded input before forwarding it into a multi-head attention layer. Its output is added to the original embedded input through a residual connection. After that, another layer normalization is applied and forwarded to a multi-layer perceptron composed of two linear layers with a Gaussian error linear units (GELU) activation between them. After another residual connection, the output of the layer is used as input for the next layer. The *L* layer’s output is summarized and the resulting vector is forwarded through a layer normalization and a simple feed-forward layer. Dropout is applied through all the processes, after every feed-forward layer, except for the last one. The diagram of this architecture is shown in Figure 2b;
- **Vanilla transformer encoder**: using the original proposed encoder in [7]. The main difference with the previous one is the application of layer normalization. Instead of applying it to embedded input, it is applied to the output of the second residual connection. The diagram of this architecture is shown in Figure 2c. Another difference is the usage of ReLU activation instead of GELU in the multi-layer perceptron.

Before forwarding features into the encoder, a linear embedding is applied to the raw input $x_{0},\ldots ,x_{N-1}$. This embedding consists of a feed-forward layer.

To account for the order of input data, a positional encoding vector $p_{0},\cdots ,p_{N-1}$ is added to each position of the previous embedded sequence $e_{0},\ldots ,e_{N-1}$. We have experimented with two possibilities: ViT encoder uses a learned encoding initialized randomly, sampled from the standard normal distribution (Equation (1a)); vanilla encoder uses a fixed positional encoding as in [7] (Equation (1b)). In the equation, pos refers to the position in the sequence, from 1 to *N*, and *i* corresponds to the position in the embedding dimension, from 0 to *d*.

(1a) P∼N(0,1)

(1b) $$P=\left\{\begin{array}{c} sin(\frac{pos}{10000^{\frac{2i}{d}}}),i\;\mathrm{mod}\;2=0\\ cos(\frac{pos}{10000^{\frac{2i}{d}}}),i\;\mathrm{mod}\;2=1 \end{array}\right.$$

The ViT encoder also includes a class token *c*, which is concatenated to the embedded output of positional encoding. This token is a learnable parameter whose purpose is to represent an aggregate sequence representation for classification tasks. Positional encoding is also applied to it ($p_{c}$). A detailed diagram of this embedding block is displayed in Figure 2a.

The output dimension of the transformer encoder is the same as the input one. Two different methods are applied to the sequence to summarize the temporal dimension: averaging over the temporal dimension and flattening the output logits into a one-dimensional vector.

#### 3.2.3. Feature Fusion Block

A feature fusion block is needed for joining the information of both branches of the model to attain a final unique prediction. Two different alternatives for this block are tested in the architecture:

- **Concatenation through fully connected**: The output of the video encoder is concatenated with the output of the kinematics encoder. This is forwarded through a multi-layer perceptron with one hidden layer, dropout regularization, and a ReLU activation. The output dimension of this layer corresponds to the number of classes;
- **Modality attention**: An attention mechanism is used to weigh the outputs of both encoders. This attention mechanism is presented in [35] and also used in PCPA model in the benchmark [8].

### 3.3. Training

This section briefly describes the datasets used and explains in more detail the loss function used, the optimizer, the training schedule, and the hardware and software used for training and testing.

#### 3.3.1. Datasets

We used the three datasets proposed in the benchmark [8] for training and testing. All of them are imbalanced with the non-crossing class being more represented than the crossing one, except for the ${\mathrm{JAAD}}_{\mathrm{beh}}$:

- **JAAD** [9]: This dataset is composed of 346 short clips (only 323 are used, excluding low resolution and adverse weather or night ones) recorded in several countries using different cameras. Two variants of the annotations are used in the benchmark: ${\mathrm{JAAD}}_{\mathrm{beh}}$ and ${\mathrm{JAAD}}_{\mathrm{all}}$. ${\mathrm{JAAD}}_{\mathrm{beh}}$ includes only pedestrians with behavioral annotations: 495 crossing and 191 non-crossing, giving rise to 374 non-crossing and 1760 crossing samples. ${\mathrm{JAAD}}_{\mathrm{all}}$ comprises the entire set of pedestrians in the sequences, adding 2100 non-crossing pedestrian far from the road, resulting in 6853 non-crossing and 1760 crossing sequence samples. The sequence samples from pedestrian tracks are extracted using a sliding window approach with an 80% of overlap between them. Bounding boxes are manually annotated and provided for each pedestrian track. However, ego-vehicle state information is not measured, and the only related annotation is the categorical ego-vehicle state. Since this annotation is not used in the original results in the benchmark, we decided not to include it in our ranking process;
- **PIE** [26]: This dataset is composed of a continuous recording session in Toronto, Canada, spanning 6 hours during the day in clear weather. In addition to bounding boxes, as in the case of JAAD, PIE provides real measurements of the ego-vehicle state, obtained using an On-Board Diagnostics (OBD) sensor. As in the case of the benchmark, we decided to include this information as input for some of the trained models in both experiments and the ranking process. It contains 512 crossing and 1322 non-crossing pedestrians, which leads to 3576 non-crossing and 1194 crossing samples, using an overlap of 60%.

#### 3.3.2. Loss

Imbalance is present in the binary classification problem we are trying to solve. To avoid that the model always predicts the majority class, we have included a weighting strategy to simulate an oversampling of the minority class, applied to the cross-entropy loss. This weight is calculated as indicated in Equation (2), where *S* corresponds to the total number of samples in the dataset, *C* refers to crossing class, and NC to non-crossing class.

(2) $$\begin{array}{c} w_{C}=S_{NC}/S,\\ w_{NC}=S_{C}/S,\\ S=S_{C}+S_{NC} \end{array}$$

The loss function is detailed on Equation (3). *x* refers to the logits or raw outputs of the network for a sequence, composed of two values, one for each class. *c* is the ground truth class for the current sequence which could be 0 or 1 (not crossing and crossing, respectively). $w_{c}$ is the weight of the ground truth class. $x_{c}$ is the raw output for the ground truth class. Finally, the index *j* traverses the interval [0,1], representing all of the classes.

(3) $$\mathrm{loss}\left(x,c\right)=-w_{c}\log\left(\frac{\exp(x_{c})}{\sum_{j}\exp\left(x_{j}\right)}\right)$$

#### 3.3.3. Optimizer

We have used AdamW optimizer [36] with a fixed learning rate whose value depends on the experiment. A scheduler is also activated which reduces the learning rate by a factor of 10 if the validation loss does not improve.

To accelerate the experimentation, we included an early stopping callback, focused on validation loss, for an early over-fitting detection during training. We perform a validation epoch every 25% of a training epoch, to have better control and avoid unnecessary computing.

#### 3.3.4. Hardware and Software Details

All the experiments using TimeSformer and most of those using RubiksNet have been performed using a single NVIDIA A100 GPU with 40 GB of memory and an AMD EPYC 7742 64-Core CPU @ 2.25 GHz.

We have chosen to use PyTorch Lightning [37] for all the experiments. This software is a high-level interface for PyTorch deep learning framework [38]. For experiment tracking and visualization of results, we have used Weights and Biases [39].

## 4. Experimental Setup

Before validating our proposed model in the previously discussed benchmark, we performed an ablation study concerning data pre-processing techniques. The objective of this study is the analysis of the importance of each input feature and also the importance of the pre-processing strategy. After this analysis, we have performed a set of experiments to see the importance of combining data for training and the generalization ability of our models. Concerning the model architecture, we performed an ablation study for video backbones and transformer encoder architectures. Finally, we evaluated the performance of our models in the benchmark.

### 4.1. Data Ablation Study

We performed several experiments concerning the input data to measure the importance of each feature and the pre-processing method applied to each of them. In addition, to measure generalization capabilities, we performed a study of the improvement achieved by our model if trained on the combination of both datasets in the benchmark.

#### 4.1.1. Bounding Boxes Image Cropping Strategies

**Figure 3:**
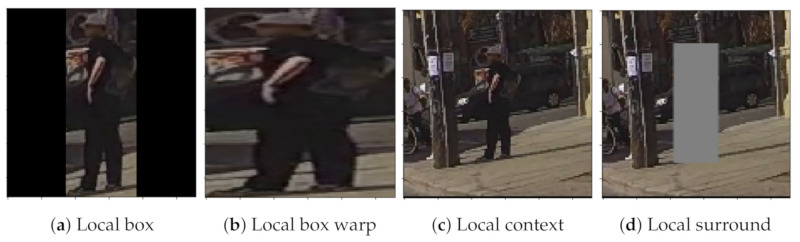
Visual example of all types of bounding boxes cropping strategies.

In the benchmark, bounding boxes coordinates are used to crop the pedestrian region from the full image frame, following different strategies: *local box*, where the bounding box is cropped and padded (black regions) to maintain the aspect ratio; *local context*, where the longest edge on the image is enlarged by a factor and the shortest is also enlarged to reach a square shape; *local surround* is the same as the previous one but deleting the image information inside the bounding box of the pedestrian and replacing it with gray pixels. Figure 3 shows a visual example of each cropping strategy.

In our experiments, we included a fourth strategy which we called *local box warp* (Figure 3b). This approach is similar to the *local box* one, but without keeping aspect ratio and, therefore, without including padding. We propose this alternative because we think it will benefit the learning process, saving the network from having to learn to ignore padded regions.

In addition to cropping strategies, we experimented with different input sizes for the video backbone. In the benchmark, the video model uses images of size 112×112×3, obtained from the cropped image which is resized accordingly. However, we experimented with a higher resolution input of 224×224×3 to see if it benefits the model’s performance.

#### 4.1.2. Bounding Box Coordinates Preprocessing

The 2D image coordinates of pedestrian bounding boxes are extracted from ground truth annotations. In the original benchmark, these coordinates, corresponding to the top-left $\left(x_{tl},\;y_{tl}\right)$ and bottom-right $\left(x_{br},\;y_{br}\right)$ coordinates, are used directly, without any normalization procedure or indirectly, through its 2D speed, obtained by subtracting to each 2D coordinate the previous one in the sequence, keeping a sequence of N−1 elements. In our case, we opted to normalize them using the min–max approach (Equation (4)) with the minimum value for both coordinates being 0 and the maximum being 1920 and 1080 for *x* and *y* image coordinates, respectively, which is the dimensions of the frames in both datasets.

(4) $$v=\frac{v-v_{min}}{v_{max}-v_{min}}$$

Additionally, we performed experiments with two variants of these coordinates (also min–max normalized):

- **Center coordinates and height**: we obtain the center coordinates of the bounding box and its height. We did not include the width to avoid redundancy, as we include it as a measure related to the distance between ego-vehicle and pedestrian;
- **Center coordinates and height including speed**: in addition to the above features, we include the speed of change of center coordinates and height.

#### 4.1.3. Pose Keypoints Missing Data

Pose keypoints provided in the benchmark are obtained offline from a convolutional pose estimation method. Since it is an error-prone method, we want to evaluate whether this input feature is helpful in any way.

#### 4.1.4. Ego-Vehicle Speed Controversy

We did not include ego-vehicle speed in our data experiments, only in the benchmark evaluation for a fair comparison with state-of-the-art models. We hypothesize that this feature is highly correlated with pedestrian action, as pedestrians tend to cross when the ego-vehicle stops and wait when the ego-vehicle does not stop. Because of that, a reduction in the ego-vehicle speed triggers the crossing action in most of the positive cases and it happens before the pedestrian decides to cross. For this reason, ego-vehicle speed should be treated as an output of an AV and not as an input to one of its perception modules.

#### 4.1.5. Input Features Combinations

Due to the ego-vehicle speed controversy and the automated nature of pose keypoints, we performed a grid analysis of all the possible combinations of input variables.

#### 4.1.6. Data Augmentation Applied

Under the hypothesis of the scarcity of training data in comparison with other general action datasets, we experimented using three types of data augmentation for video sequences. If the augmentation is applied, the same change is performed in all images of a sequence:

- **Horizontal flip**: apply a random horizontal flip on the image plane;
- **Roll rotation**: apply a roll rotation of the 3D sequence, which is equivalent to applying a 2D rotation on each image in the sequence;
- **Color jittering**: apply a random change in brightness, contrast, saturation, and hue of the input sequence.

#### 4.1.7. Combined Datasets Training

Deep learning models perform better with more varied training data, becoming models prone to task generalization rather than over-fitting. To test this property, we performed two different training schedules: one model only trained on the PIE dataset, and another one trained on both PIE and JAAD. We tested both models on both datasets test sets.

### 4.2. Model Ablation Study

We performed two groups of experiments, focused on the video backbone and the transformer encoder used in the kinematics branch. For the first group, we explored different variants of RubiksNet and TimeSformer, pre-trained on different datasets. In the case of the transformer encoder, we experimented with different encoder types and different summarizing strategies.

#### 4.2.1. Pre-Trained Backbones

For RubiksNet backbone, we have used a variety of pre-trained weights, ranging from RubiksNet-Tiny variant (1.9 M parameters and 3.9 GFLOPs) to RubiksNet-Large variant (8.5 M parameters and 15.8 GFLOPs). We also experimented with different pre-training datasets: Something-Something-V2 (SSv2) [40] and Kinetics-400 (K400) [41] datasets. TimeSformer pre-trained models are notably larger. We selected the smaller model with 121.4 M parameters and 590 GFLOPs at inference. We also experimented with the previous pre-training datasets and additional ones: HowTo100M [42] (HT100M) and Kinetics-600 [43] (K600).

After several experiments with all of the above-mentioned backbones, we found that SSv2 pre-trained models outperformed the rest by a large margin. One of the possible reasons is the training schedule followed with the rest of the datasets. We also trained the backbone from scratch, but SSv2 pre-trained model also outperforms this strategy. For this reason, we have chosen this backbone in all our experiments.

#### 4.2.2. Different Transformer Encoders

Two different transformer encoder architectures are proposed in this work. In addition, its output is processed using the mean operation over the temporal dimension or by applying a flattening operation, resulting in a one-dimensional vector. By combining the encoder type and the output summarizing strategy, we obtained four different options. The best performing option is also compared with the model without a kinematic branch, to see if this branch improves the result.

### 4.3. Benchmark

After performing all of the previous experiments, we have found which pre-processing strategies improve our model’s performance and also the importance of the different input features.

We have compared our model variations with four different models ranked as the best-performing ones in the benchmark:

- **Multi-stream RNN (MultiRNN)** [44]: Two stream architecture which combines two RNN streams, one for odometry prediction (includes a CNN encoder for including visual features) and the other for bounding box prediction. Instead of predicting future bounding boxes, it is modified to predict the future pedestrian crossing action;
- **C3D** [45]: 3D convolutional model which combines 3D convolutional layers and 3D max-pooling layers. It uses only the pedestrian bounding box cropped regions from RGB video sequences as input data;
- **Inflated 3D (I3D)** [41]: 3D convolutional model based on 2D CNN inflation, where filters and pooling kernels are expanded into 3D. It uses as input optical flow sequence information, extracted from pedestrian bounding box cropped regions;
- **PCPA** [8]: best performing model in the benchmark. Multi-branch model with four branches. The first branch is based on C3D network and encodes input RGB video sequence extracted by cropping pedestrian bounding boxes. The other three branches consist of RNNs. The information is fussed using attention at the temporal level and the branch (modality) level.

However, for a fair comparison with PCPA, the best model in the benchmark [8], we have used the same pre-processing strategy it followed. Using TimeSformer and RubiksNet backbones, we experimented with the use of modal attention or concatenation as fusion block of the output of the different branches and we have used the findings from the ablation study of the model.

To check if this performance similitude is due to the transformer encoder and not to the video backbone, we developed our best transformer encoder in Tensorflow and changed the recurrent encoders in the PCPA model with it. All non-image (kinematics) inputs are concatenated and forwarded through it after embedding them. The rest of the PCPA model remains the same, including the video 3D backbone and the fusion part. However, we changed the optimizer to AdamW and tuned the hyperparameters of our model to accelerate training. As AdamW applies weight decay, we deactivated direct regularization on layers and applied a weight decay of $10^{-4}$ for all experiments. We also performed all experiments only using image bounding boxes crops and coordinates, following *local box* cropping strategy and without any normalization, respectively. Our combined features transformer encoder have the following hyperparameters:

- Query, key and value size is the same $d_{q,k,v}\equiv d=256$;
- Number of self-attention heads $n_{heads}=8$;
- Number of transformer encoders L=2;
- Multi-layer perceptron hidden layer dimension $d_{mlp}=384$;
- Dropout rate, applied after embedding and the MLP block $p_{drop}=0.1$.

### 4.4. Model Hyperparameters

Unless specified, we performed all of the previous detailed experiments with the same hyperparameters, to see the effects of data modification on the results. These experiments are not focused on reaching the best performing metrics but on reaching average results in a short period. This default set of hyperparameters is the following:

- PIE dataset used for training;
- Batch size of 16 samples;
- *Local box warp* used as the pre-processing for bounding boxes crops;
- TimeSformer used as backbone, pre-trained on SSv2 and fine-tuned. The output vector size of 1024;
- Fusion strategy: concatenation;
- Input sequence length N=16;
- Learning rate with value $10^{-4}$ for the fusion and kinematic encoder and $10^{-5}$ for the video backbone;
- Input image dimension of 112×112×3;
- Weight decay of $10^{-3}$.

Due to the difficulty of the task given and the stochastic nature of models and training algorithms, we gather results of every experiment using a set of eight different random seeds, displaying on result tables the mean and its standard error, to show the discrepancy between the group of eight samples and the real distribution mean.

For the benchmark models, we fixed random seed to 42 in both PyTorch and Tensorflow implementation. We have included as Supplementary Material  the code for Tensorflow model.

### 4.5. Metrics

We have used the same metrics available in the benchmark. Accuracy does not represent a good performance estimator in imbalanced problems, so we focused our analysis of the results on F1 score (F1) and the area under the Receiver Operating Characteristic (ROC) curve (AUC). F1 Score is the harmonic mean of precision and recall. It is used as a measure in which both metrics have the same weight. However, in real applications, higher recall is more important to improve safety and higher precision is better to avoid false positives and allow smoother driving, decreasing unnecessary braking. For this reason, we have also included precision and recall. Finally, AUC represents the ability of our classifier to distinguish between both classes. A value of 0.5 means that the classifier behavior is equivalent to randomly choosing the class. Metrics are calculated for the positive class (crossing case) using the scikit-learn library [46].

## 5. Results

### 5.1. Preprocessing

#### 5.1.1. Image Input Nature and Size

The results for each cropping strategy in Section 4.1.2 are shown in Table 2. Keeping aspect ratio seems prone to over-fitting. This procedure obtains good results in the original benchmark work because it uses a considerably lower learning rate and a bigger number of epochs. However, in the case of the rest of cropping strategies proposed in the benchmark, the results are much better, nearly doubling F1 score. Finally, our proposed strategy seems to be the best performing one using our learning strategies, obtaining the best results in all of the metrics.

With respect to the input size (results on Table 3), benchmark’s model video backbone uses 112×112. However, TimeSformer is pre-trained using four times bigger images of 224×224. Training the model with an image with lower resolution seems to affect negatively, even when the video backbone is fine-tuned. Higher details in input image, both from the pedestrian and from the minimal context included in the *local box* cropping strategy, are better for the results than applying data augmentation (detailed in Section 5.1.4). One drawback of this approach is the memory consumption, which reaches ≈39 GB with a batch size of 16 samples.

#### 5.1.2. Bounding Box Coordinates Preprocessing

Among the three pre-processing strategies applied to the bounding box coordinates, only the one using center coordinates and height obtains better results than the only-image case (see Table 4). The recall is lower compared to the only-image case. Precision is higher in all cases except for the last one, which includes the speed of coordinates. The image plane localization information provided by bounding boxes coordinates could help the network discriminate samples with similar image information (e.g., a pedestrian walking near the road but not crossing from one approaching the road with the intention of crossing). However, this feature does not provide a clear improvement in the performance of our model.

#### 5.1.3. Different Combinations of Input Features

In Table 5 we can see the results of training the model with different input features. Although the combination of all input features proposed in the benchmark obtains good results, we can find an improvement by leaving pose keypoints out of the input set. These keypoints are precomputed by a pose estimation model instead of hand-labeled, which leads to data with a high percentage of missing values which represent 80.9/78.5/84.4% on the JAAD dataset and 44.5/31.0/23.6% on the PIE dataset (train, test, and validation, respectively). Its negative effect reaches the maximum level when it is used alone, where the network does not find any information in it during training. In contrast, bounding box coordinates improves the results slightly from randomness.

**Figure 4:**
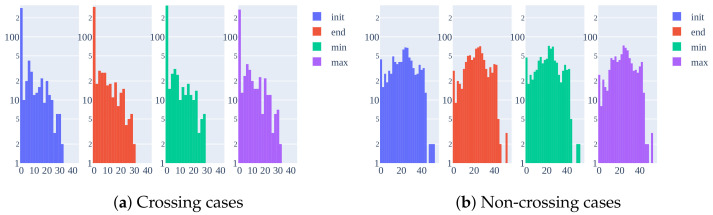
Histogram showing the number of pedestrian tracks for each range of ego-vehicle speed in the benchmark data (TTE from 30 to 60 frames).

Ego-vehicle speed is the feature with more weight in the results. Every combination where it is included, F1 increases by more than 25%. In PIE dataset, the ego-vehicle speed is obtained using an OBD sensor installed in the vehicle. Only pedestrians who can interact with the driver have behavioral annotations. This is a major drawback as it is shown in the histogram in Figure 4a. Most of the crossing cases correspond to a low speed near zero because the ego-vehicle lowers the speed or even stops when a pedestrian crosses unless it is far away on the ego-lane, which is not usual. In the non-crossing scenario (Figure 4b), the ego-vehicle speed has a more uniform distribution. Both figures represent the histogram at the first sample in the benchmark time interval (init=TTE−60) and in the last sample (end=TTE−30), including the minimum and maximum values in that interval.

This differentiation between both classes converts this input feature into the most valuable one, outperforming alone even the combination proposed in the benchmark. A model trained with this feature ends learning the behavior of the ego-vehicle driver instead of learning to anticipate the behavior of pedestrians.

#### 5.1.4. Data Augmentation

We applied data augmentation with two different probabilities: an aggressive one of 50% and a milder one with 25%. Aggressive policy results are shown in Table 6. In the case of using only the video backbone, rotation is the most valuable augmentation, increasing F1 by nearly a 5% and AUC a 2%. However, with the inclusion of bounding box coordinates, the performance using rotation drops. Bounding box coordinates are not modified because the center of rotation is performed using the center of the bounding box coordinates. The height is also rotation invariant. However, this drop in performance can be caused by showing during training different sources of images with the same bounding box coordinates sequences. However, this hypothesis is rejected in the case of the horizontal flip, where, even applying a flip transformation to bounding box coordinates, the performance still drops in comparison to the case of using only video as input. On the other hand, color jittering has the opposite effect: improves results using both inputs and performance decays when only uses the image.

Individually, all transformations behave differently, but applying all three improves the results in all combinations of input data, showing that the best possible strategy is the combination.

Finally, the mild strategy of 25% is not enough to improve the results concerning the non-augmented version. For this reason, we did not include another table with its results.

### 5.2. Combined Datasets Training

Although the PIE dataset is quite diverse in pedestrian behaviors, as it is shown in Table 7, training only with PIE does not generalize to the JAAD dataset. This could be caused by the more variety in weather and light since PIE is continuously recorded on the same day and same city under clear weather.

Only by combining training sets of both datasets, the model seems to generalize to both of them, achieving slightly worse results in PIE and a major improvement in JAAD.

### 5.3. Different Encoder Strategies

Looking at the results shown in Table 8, performing the mean operation over the temporal domain seems to be the best strategy. Using flattening we obtain worse results than in the only-image case (first row). Vanilla transformer with predefined positional encoding performs better than ViT transformer in this case, where this encoding is learned during training. It is especially important the difference in recall metric due to the criticality of obtaining false negatives in the crossing class on the road scenario.

However, since this experiment is part of a data ablation study, we are confident in using the mean as the best strategy, but not in the best encoder type, since both encoders have the same hyperparameters and we cannot assure that those are equally beneficial for both architectures.

### 5.4. Benchmark

In Table 9 we gathered the results obtained with our best performing models. In the case of TimeSformer, we attained slightly better results with concatenation fusion. Models were trained using 112×112 images and half of the input video sequence (N/2=8). In addition, we did not include pose information while training, which makes it unnecessary to include a pose estimation model in the prediction pipeline.

The main problem of TimeSformer is its considerable size (a total ≈123 million parameters for the whole CAPformer). As a lighter alternative, we performed experiments with the RubiksNet tiny version, which in combination with the rest of the model, contains only 3.5 and 4 million parameters in the modality attention and concatenation variants, respectively. RubiksNet case is also lighter than C3D backbone, which depending on the number of input kinematic features, englobes a total amount of ≈31 million parameters. This dependence is due to the use of one recurrent encoder for each input kinematic feature.

To see if the transformer encoder for kinematics features was indeed the reason for these good results, we included this encoder in the PCPA model, substituting recurrent ones. We trained these models with only bounding boxes image crops and coordinates, obtaining comparable results to the ones obtained with TimeSformer and RubiksNet, and also with some of the PCPA variants. For a fair comparison, we trained two PCPA variants with the same random seed used in our model. The first case uses the best-performing model in the benchmark and the same training procedure. We can see that there is a big difference between the final results (F1=0.770 vs. F1=0.735) in PIE. It is quite noticeable the big difference obtained in the ${\mathrm{JAAD}}_{\mathrm{all}}$ dataset. In ${\mathrm{JAAD}}_{\mathrm{beh}}$ dataset, C3D, I3D, and MultiRNN obtain better F1 score than our method. However, looking at the AUC, they are closer to 0.5, meaning that its behavior is more random than in our method. In summary, looking at the results obtained, our method performs better than PCPA model, validating our proposed encoder based on self-attention mechanism rather than recurrent sequential ones.

Finally, as it is shown by the results, there is a big dependency on the randomness of the data. With a different random seed to the one used in the results in Table 9, we obtained F1=0.807 and AUC=0.922 with the same RubiksNet tiny backbone. Additionally, by training the PCPA model, we obtained without fixing the random seed a model with F1=0.794 and AUC=0.875, which means that randomness considerably affects the performance of the network. This could be caused by data scarcity. Even though JAAD and PIE are two complete datasets with high-quality annotations, the variety in them could not be enough for a model using video as input to generalize. Another possible reason could be the complexity of the task since samples from both classes have similar image features in the prediction interval proposed by the benchmark.

### 5.5. Qualitative Results

**Figure 5:**
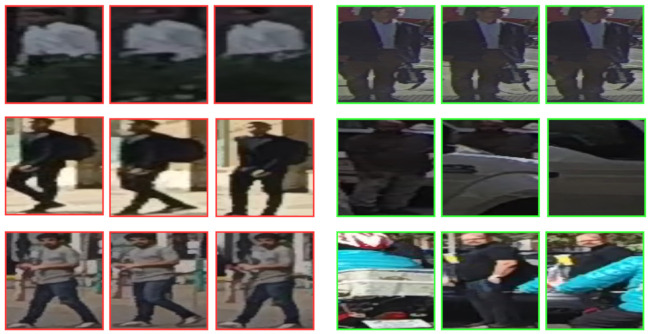
Correct predictions in different test cases obtained from RubiksNet trained model. Green and red borders represent crossing and not crossing behavior, respectively.

**Figure 6:**
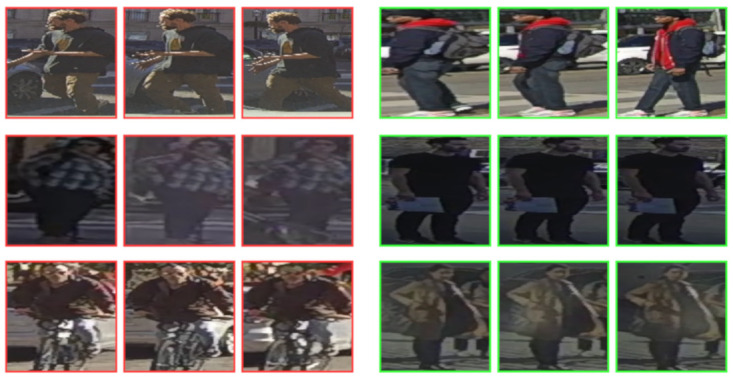
Incorrect predictions in different test cases obtained from RubiksNet trained model. Green and red borders represent crossing and not crossing behavior, respectively.

In addition to the quantitative results shown in the previous section, qualitative results can help to better understand the behavior of the system by analyzing correct (Figure 5) and incorrect (Figure 6) predictions obtained from RubiksNet trained model. Each case is represented by three images inside the sequence (first, middle, and last). Among correct cases, we can observe that the network can predict crossing cases with 2 s of anticipation with strong occlusion from cars (middle-right) and cyclists (bottom-right). In the top-right case, the network is able to predict a difficult case, where the pedestrian remains stationary for most of the time. The network helps with the positional knowledge and possibly with the looking state of the pedestrian. Non-crossing cases are also correctly classified even with the occlusion of bushes (top-left). In the middle-left case, the pedestrian approaches the road perpendicularly, however it is correctly classified since he slows down his walking pace to a near standstill. A similar case is shown in the bottom-left, showing that the network can generalize to different pedestrians and scenes and is able to focus on motion information.

Among failure cases (Figure 6) there are also interesting situations. The top-right crossing case is wrongly predicted at the beginning of the track with a similar probability for both classes. However, the correct class is predicted with a 1 s anticipation (last sample). The middle-left case is wrongly detected as crossing, probably because the information obtained from input data can indicate the pedestrian’s intention to cross. Difficult cases are shown also in middle-right and bottom-right cases, where the situation is difficult to interpret even for a driver. The network obtains a similar probability for both classes. In the first case, this similarity between both classes disappears after two sequences, when the correct class is predicted by the network. Nevertheless, in the second case, the uncertainty remains for all samples of the target pedestrian. Finally, we have found different cases which are included by error (cyclist on bottom-left) or incorrectly labeled (pedestrian walking through cars labeled as not crossing) on the top-left. These inconsistencies are detrimental to network learning if they are present in the training set and to the performance evaluated in tests.

## 6. Discussion

Pedestrian crossing action prediction is a complex task even for humans. The results obtained in the benchmark show that there is a big dependency on randomness, possibly due to data scarcity and complexity of the data, which includes small and occluded pedestrians. Relying on image data as the primary source of information exacerbates the problem of data scarcity. Deep learning techniques usually improve their generalization and avoid over-fitting if more data and more variety are used for training. Training with both datasets shows a generalization to both test sets. More varied data from different countries, with different weather and light conditions, could be the main option for improving the results, rather than focusing on the optimization of hyperparameters in the models available. Although we reach good results in the benchmark, we are using ground truth information, assuming ideal object detection and tracking. Incorporating the noise of these modules could highlight problems in current architectures.

Another possible way to improve the performance of the model could be the usage of lower-dimensional data. Instead of including raw image data in the equation, semantic information could lead to better results as is the case of [30]. Including 3D information could be another valuable source. However, currently, this option is not available in the available pedestrian behavior datasets. With the release of PePScenes [27], these features will become available.

Pose information degrades the performance of our models. This finding reinforces the fact that one of the main strategies to be followed to attain better results is the improvement of data quality.

It is important to notice the change in results when ego-vehicle speed is used as input in the estimation of the crossing behavior. This variable can distort the results, preventing a generalization towards cases where speed has less differentiated values, such as cases of risk of collision due to jaywalking. It is a rare situation where a pedestrian decides to cross if the vehicle has not started braking or is stationary. Even with the amount of work in the labeling of both datasets, JAAD and PIE do not contain enough of these edge cases and even in a predefined environment, it would be dangerous to perform this group of experiments. One possible solution is the use of simulators, such as CARLA [47], in combination with virtual reality systems to include real behaviors in autonomous driving simulators, which would allow the generation of multiple critical and edge cases without safety concerns.

## 7. Conclusions and Future Work

In this work, we have proposed an alternative to recurrent approaches for pedestrian crossing action anticipation. We focused on the pre-processing of the data, performing several experiments during which we found problems with ego-vehicle speed and pose keypoints, not highlighted in the benchmark. In the last part of our analysis, we performed an evaluation of different variants of our model in the benchmark proposed in [8] reaching comparable or better results using fewer input data. These findings validate our new approach and also rise to a reformulation of some benchmark conditions, mainly related to pre-processing. From a practical point of view, good results in this task can lead to an improvement in the autonomous vehicle perception pipeline, where only pedestrians who are willing to cross will be considered. This filtering step helps to reduce the computational cost of the scene perception and allows the vehicle to focus better on critical situations. Even without a computational improvement, since it does not require prior mapping, this system could be implemented to improve pedestrian detection systems through anticipation, which drastically reduces the probability of serious injury or death in urban collisions.

In the future, some possible lines of work are detailed below:

- Evaluate our model in additional datasets, such as STIP [25] or TITAN [24];
- Train and test models in a combination of different available behavior datasets and analyze it in an unrelated scenario to see its generalization capabilities;
- Research deeper in the usage of data augmentation techniques in the training of multi-branch models;
- Consider the development of virtual scenarios to include more crossing cases and fight data imbalance;
- Explore new features from datasets, such as labeled information from the environment (e.g., the relative relationship between vehicles, crosswalk position, traffic signals). These new features will be 2D or 3D, depending on the availability of datasets in the literature;
- Experimentation with different data cleaning strategies in training time, applying a maximum occlusion level, pedestrian minimum size, etc.;
- Simulate real case scenario to find new weaknesses and strengths in available models.

## Figures and Tables

**Table:** 

0.325	0.325	0.325
(a) b	(b) b	(c) b

**Table:** 

0.24	0.24	0.24	0.24
(a) b	(b) b	(c) b	(d) b

**Table:** 

0.49	0.49
(a) b	(b) b

**Table 1:** Information about the two datasets included in the benchmark. # of ann. fr. refers to the number of annotated frames; $S_{\mathrm{NC}}$ and $S_{C}$ refers to the number of non-crossing and crossing pedestrians, respectively; Ego-veh. mot. refers to ego-vehicle motion.

	# of ann. fr.	$S_{\mathrm{NC}}$	$S_{C}$	Diff. Weather	Diff. Loc.	Ego-Veh. Mot.
**JAAD**	75 K	495	2291	Yes	Yes	No
**PIE**	293 K	512	1322	No	No	Yes

**Table 2:** Results obtained varying input image nature.

	F1	P	R	AUC
box	0.212±0.078	0.309±0.081	0.189±0.069	0.542±0.014
box warp	0.454±0.040	0.532±0.015	0.417±0.055	0.636±0.019
context	0.387±0.017	0.531±0.037	0.318±0.025	0.599±0.006
surround	0.379±0.070	0.431±0.072	0.350±0.071	0.607±0.022

**Table 3:** Results obtained varying input image size.

	F1	P	R	AUC
112×112	0.454±0.040	0.532±0.015	0.417±0.055	0.636±0.019
224×224	0.528±0.020	0.572±0.021	0.507±0.042	0.636±0.011

**Table 4:** Results obtained using different pre-processing strategies for bounding box coordinates. *h* refers to bounding box height; $x^{\prime },y^{\prime },h^{\prime }$ refers to speed of x,y coordinates and height, respectively; subscripts tl,br,c refers to top-left, bottom-right and center coordinates of the bounding box.

Mode	F1	P	R	AUC
Only image	0.454±0.040	0.532±0.015	0.417±0.055	0.636±0.019
$x_{tl},y_{tl},x_{br},y_{br}$	0.425±0.023	0.550±0.020	0.358±0.035	0.620±0.011
$x_{c},y_{c},h$	0.456±0.031	0.587±0.031	0.403±0.052	0.639±0.013
$x_{c},y_{c},h,x_{c}^{\prime },y_{c}^{\prime },h^{\prime }$	0.355±0.067	0.462±0.078	0.300±0.066	0.600±0.022

**Table 5:** Different input combinations metrics. Abbr. I: Bounding boxes image crops, B: bounding boxes coordinates, P: pose keypoints, S: ego-vehicle speed.

Input				F1	P	R	AUC
I	B	P	S				
	🗸			0.160±0.049	0.428±0.115	0.108±0.038	0.535±0.011
	🗸	🗸		0.011±0.007	0.204±0.140	0.006±0.004	0.502±0.002
	🗸	🗸	🗸	0.737±0.004	0.660±0.011	0.837±0.013	0.833±0.003
	🗸		🗸	0.746±0.003	0.698±0.017	0.806±0.016	0.833±0.002
🗸				0.454±0.040	0.532±0.015	0.417±0.055	0.636±0.019
🗸	🗸			0.456±0.031	0.587±0.031	0.403±0.052	0.639±0.013
🗸	🗸	🗸		0.454±0.030	0.533±0.020	0.403±0.039	0.633±0.015
🗸	🗸	🗸	🗸	0.716±0.010	0.695±0.016	0.748±0.032	0.808±0.010
🗸	🗸		🗸	0.726±0.007	0.665±0.014	0.803±0.017	0.821±0.006
🗸		🗸		0.468±0.022	0.542±0.013	0.420±0.034	0.640±0.011
🗸		🗸	🗸	0.726±0.009	0.701±0.012	0.759±0.028	0.815±0.009
🗸			🗸	0.701±0.013	0.685±0.017	0.731±0.040	0.798±0.013
		🗸		0.000±0.000	0.000±0.000	0.000±0.000	0.500±0.000
		🗸	🗸	0.734±0.002	0.644±0.004	0.854±0.004	0.834±0.001
			🗸	0.743±0.003	0.680±0.010	0.819±0.011	0.834±0.002

**Table 6:** Results of different data augmentation strategies. Abbr. C: Color jittering, F: horizontal flip (left to right), R: 2D rotation (roll angle), I: image bounding box crop, B: bounding box coordinates.

Augm.	Input	F1	P	R	AUC
−	I	0.454±0.040	0.532±0.015	0.417±0.055	0.636±0.019
	I+B	0.456±0.031	0.587±0.031	0.403±0.052	0.639±0.013
C	I	0.446±0.032	0.572±0.020	0.385±0.050	0.634±0.016
	I+B	0.473±0.016	0.544±0.011	0.422±0.022	0.641±0.008
F	I	0.475±0.011	0.585±0.019	0.405±0.020	0.644±0.005
	I+B	0.443±0.029	0.557±0.026	0.390±0.049	0.630±0.014
F+R+C	I	0.469±0.015	0.600±0.015	0.390±0.024	0.643±0.007
	I+B	0.513±0.025	0.562±0.019	0.484±0.044	0.667±0.015
R	I	0.504±0.012	0.567±0.017	0.458±0.018	0.659±0.007
	I+B	0.434±0.048	0.572±0.027	0.383±0.063	0.631±0.021

**Table 7:** Combined dataset training experiment. Abbr. P: PIE, J: JAAD. Train and Test columns refer to the dataset used for training and testing, respectively.

Train	Test	F1	P	R	AUC
P+J	P	0.427±0.035	0.478±0.023	0.419±0.055	0.615±0.015
	J	0.566±0.020	0.540±0.026	0.635±0.055	0.756±0.018
P	P	0.454±0.040	0.532±0.015	0.416±0.068	0.636±0.019
	J	0.207±0.021	0.171±0.012	0.275±0.043	0.500±0.013

**Table 8:** Different transformer encoder configurations experiment. Abbr. mean: output average strategy, flat: output flattening strategy.

		F1	P	R	AUC
−	−	0.454±0.040	0.532±0.015	0.417±0.055	0.636±0.019
ViT [34]	mean	0.456±0.031	0.587±0.031	0.403±0.052	0.639±0.013
	flat	0.415±0.042	0.532±0.029	0.352±0.048	0.617±0.020
Vanilla [7]	mean	0.519±0.014	0.557±0.019	0.500±0.034	0.669±0.008
	flat	0.431±0.039	0.571±0.018	0.367±0.052	0.628±0.017

**Table 9:** Comparison of our proposed model and the best-performing models in benchmark [8]. Abbr. M is modality attention; C is concatenation; T is temporal attention; I refers to bounding box image crops; B refers to bounding box coordinates; S refers to ego-vehicle speed and P refers to pose keypoints. Rows with darker background correspond to PCPA models trained by us.

Model	Backbone	Fusion	Input	PIE		${\mathrm{JAAD}}_{\mathrm{beh}}$		${\mathrm{JAAD}}_{\mathrm{all}}$	
				F1	AUC	F1	AUC	F1	AUC
Ours	TimeSformer	M	I,B,S	0.761	0.844	**0.763**	0.545	0.557	0.728
		C		**0.779**	0.853	0.743	0.552	0.514	0.701
	RubiksNet	M		0.749	0.839	0.752	**0.589**	0.630	0.782
		C		0.738	0.828	0.691	0.549	0.618	0.778
	C3D	M,T	I,B	0.750	0.851	0.615	0.577	0.614	0.802
C3D		–	I	0.520	0.670	0.750	0.510	0.650	0.810
MultiRNN	GRU	–	B, S *	0.710	0.800	0.740	0.500	0.580	0.790
I3D		–	O	0.720	0.830	0.750	0.510	0.630	0.800
PCPA	C3D	C	I,B,S,P	0.730	0.830	0.630	0.480	0.580	0.800
		M		0.750	0.840	0.680	0.490	0.620	0.830
		T		0.770	**0.860**	0.710	0.480	0.620	0.790
		M,T		0.770	**0.860**	0.710	0.500	**0.680**	**0.860**
				0.735	0.834	0.630	0.484	0.530	0.779
			I,B	0.723	0.820	0.613	0.486	0.522	0.780

* We are not sure of the data used by this network. We indicated bounding boxes coordinates and ego-vehicle speed since this is the data used in the original work.

## Data Availability

Not applicable.

## Supplementary Materials

Code and instructions for reproducing TensorFlow experiments on the benchmark are available in https://github.com/javierlorenzod/CAPformer.

## References

[1] World Health Organization (2018). Global Status Report on Road Safety 2018.

[2] Adminaité-Fodor D., Jost G. (2019). Safer Roads, Safer Cities: How to Improve Urban Road Safety in The EU.

[3] (2020). European New Car Assessment Programme (Euro NCAP) Test Protocol-AEB VRU Systems.

[4] Rudenko A., Palmieri L., Herman M., Kitani K.M., Gavrila D.M., Arras K.O. (2020). Human motion trajectory prediction: A survey. Int. J. Robot. Res..

[5] Rasouli A., Kotseruba I., Tsotsos J.K. (2020). Pedestrian Action Anticipation Using Contextual Feature Fusion in Stacked RNNs. arXiv.

[6] Zhu Y., Li X., Liu C., Zolfaghari M., Xiong Y., Wu C., Zhang Z., Tighe J., Manmatha R., Li M. (2020). A Comprehensive Study of Deep Video Action Recognition. arXiv.

[7] Vaswani A., Shazeer N., Parmar N., Uszkoreit J., Jones L., Gomez A.N., Kaiser L., Polosukhin I. (2017). Attention Is All You Need. arXiv.

[8] Kotseruba I., Rasouli A., Tsotsos J.K. Benchmark for Evaluating Pedestrian Action Prediction. Proceedings of the IEEE Winter Conference on Applications of Computer Vision (WACV).

[9] Rasouli A., Tsotsos J.K. (2018). Joint Attention in Driver-Pedestrian Interaction: From Theory to Practice. arXiv.

[10] Rasouli A., Kotseruba I., Tsotsos J.K. Are They Going to Cross? A Benchmark Dataset and Baseline for Pedestrian Crosswalk Behavior. Proceedings of the 2017 IEEE International Conference on Computer Vision Workshops (ICCVW).

[11] Fang Z., López A.M. (2018). Is the Pedestrian going to Cross? Answering by 2D Pose Estimation. arXiv.

[12] Gesnouin J., Pechberti S., Bresson G., Stanciulescu B., Moutarde F. (2020). Predicting Intentions of Pedestrians from 2D Skeletal Pose Sequences with a Representation-Focused Multi-Branch Deep Learning Network. Algorithms.

[13] Cadena P.R.G., Yang M., Qian Y., Wang C. (2019). Pedestrian Graph: Pedestrian Crossing Prediction Based on 2D Pose Estimation and Graph Convolutional Networks. Proceedings of the 2019 IEEE Intelligent Transportation Systems Conference, ITSC.

[14] Ait Bouhsain S., Alahi A. (2020). Pedestrian Intention Prediction: A Multi-Task Perspective. Technical Report. arXiv.

[15] Lorenzo J., Parra I., Wirth F., Stiller C., Llorca D.F., Sotelo M.A. (2020). RNN-based Pedestrian Crossing Prediction using Activity and Pose-related Features. Proceedings of the IEEE Intelligent Vehicles Symposium.

[16] Ghori O., MacKowiak R., Bautista M., Beuter N., Drumond L., DIego F., Ommer B.B. Learning to Forecast Pedestrian Intention from Pose Dynamics. Proceedings of the 2018 IEEE Intelligent Vehicles Symposium (IV).

[17] Ranga A., Giruzzi F., Bhanushali J., Wirbel E., Pérez P., Vu T.H., Perrotton X. (2020). VRUNet: Multi-Task Learning Model for Intent Prediction of Vulnerable Road Users. IS T Int. Symp. Electron. Imaging Sci. Technol..

[18] Pop D.O., Rogozan A., Chatelain C., Nashashibi F., Bensrhair A. (2019). Multi-Task Deep Learning for Pedestrian Detection, Action Recognition and Time to Cross Prediction. IEEE Access.

[19] Saleh K., Hossny M., Nahavandi S. (2019). Real-time Intent Prediction of Pedestrians for Autonomous Ground Vehicles via Spatio-Temporal DenseNet. arXiv.

[20] Yang B., Zhan W., Wang P., Chan C., Cai Y., Wang N. (2021). Crossing or Not? Context-Based Recognition of Pedestrian Crossing Intention in the Urban Environment. IEEE Trans. Intell. Transp. Syst..

[21] Piccoli F., Balakrishnan R., Perez M.J., Sachdeo M., Nunez C., Tang M., Andreasson K., Bjurek K., Raj R.D., Davidsson E. (2020). FuSSI-Net: Fusion of Spatio-temporal Skeletons for Intention Prediction Network. arXiv.

[22] Gujjar P., Vaughan R. (2019). Classifying pedestrian actions in advance using predicted video of urban driving scenes. Proceedings of the IEEE International Conference on Robotics and Automation.

[23] Chaabane M., Trabelsi A., Blanchard N., Beveridge R. (2020). Looking ahead: Anticipating pedestrians crossing with future frames prediction. Proceedings of the 2020 IEEE Winter Conference on Applications of Computer Vision, WACV.

[24] Malla S., Dariush B., Choi C. TITAN: Future Forecast using Action Priors. Proceedings of the 2020 IEEE/CVF Conference on Computer Vision and Pattern Recognition (CVPR).

[25] Liu B., Adeli E., Cao Z., Lee K.H., Shenoi A., Gaidon A., Niebles J.C. (2020). Spatiotemporal Relationship Reasoning for Pedestrian Intent Prediction. IEEE Robot. Autom. Lett..

[26] Rasouli A., Kotseruba I., Kunic T., Tsotsos J.K. PIE: A Large-Scale Dataset and Models for Pedestrian Intention Estimation and Trajectory Prediction. Proceedings of the 2019 IEEE/CVF International Conference on Computer Vision (ICCV).

[27] Rasouli A., Yau T., Lakner P., Malekmohammadi S., Rohani M., Luo J. (2020). PePScenes: A Novel Dataset and Baseline for Pedestrian Action Prediction in 3D. arXiv.

[28] Caesar H., Bankiti V., Lang A.H., Vora S., Liong V.E., Xu Q., Krishnan A., Pan Y., Baldan G., Beijbom O. (2019). nuScenes: A Multimodal Dataset for Autonomous Driving. arXiv.

[29] Yau T., Malekmohammadi S., Rasouli A., Lakner P., Rohani M., Luo J. (2020). Graph-SIM: A Graph-based Spatiotemporal Interaction Modelling for Pedestrian Action Prediction. arXiv.

[30] Yang D., Zhang H., Yurtsever E., Redmill K., Özgüner Ü. (2021). Predicting Pedestrian Crossing Intention with Feature Fusion and Spatio-Temporal Attention. arXiv.

[31] Fan L., Buch S., Wang G., Cao R., Zhu Y., Niebles J.C., Fei-Fei L. RubiksNet: Learnable 3D-Shift for Efficient Video Action Recognition. Proceedings of the European Conference on Computer Vision (ECCV).

[32] Bertasius G., Wang H., Torresani L. Is Space-Time Attention All You Need for Video Understanding? In Proceedings of the International Conference on Machine Learning (ICML), 18–24 July 2021.

[33] Khan S., Naseer M., Hayat M., Waqas Zamir S., Shahbaz Khan F., Shah M. (2021). Transformers in Vision: A Survey. arXiv.

[34] Dosovitskiy A., Beyer L., Kolesnikov A., Weissenborn D., Zhai X., Unterthiner T., Dehghani M., Minderer M., Heigold G., Gelly S. (2020). An Image Is Worth 16x16 Words: Transformers for Image Recognition at Scale. arXiv.

[35] Yang Z., Yang D., Dyer C., He X., Smola A., Hovy E. Hierarchical attention networks for document classification. Proceedings of the 2016 Conference of the North American Chapter of the Association for Computational Linguistics: Human Language Technologies (NAACL HLT).

[36] Loshchilov I., Hutter F. (2019). Decoupled Weight Decay Regularization. arXiv.

[37] (2019). Falcon, WA, e.a. PyTorch Lightning. GitHub. https://github.com/PyTorchLightning/pytorch-lightning.

[38] Paszke A., Gross S., Massa F., Lerer A., Bradbury J., Chanan G., Killeen T., Lin Z., Gimelshein N., Antiga L., Wallach H., Larochelle H., Beygelzimer A., d’Alché-Buc F., Fox E., Garnett R. (2019). PyTorch: An Imperative Style, High-Performance Deep Learning Library. Advances in Neural Information Processing Systems 32 (NeurIPS).

[39] Biewald L. (2020). Experiment Tracking with Weights and Biases. wandb.com.

[40] Goyal R., Kahou S.E., Michalski V., Materzyńska J., Westphal S., Kim H., Haenel V., Fruend I., Yianilos P., Mueller-Freitag M. (2017). The “Something Something” Video Database for Learning and Evaluating Visual Common Sense. arXiv.

[41] Carreira J., Zisserman A. (2018). Quo Vadis, Action Recognition? A New Model and the Kinetics Dataset. arXiv.

[42] Miech A., Zhukov D., Alayrac J.B., Tapaswi M., Laptev I., Sivic J. HowTo100M: Learning a Text-Video Embedding by Watching Hundred Million Narrated Video Clips. Proceedings of the 2019 IEEE/CVF International Conference on Computer Vision (ICCV).

[43] Carreira J., Noland E., Banki-Horvath A., Hillier C., Zisserman A. (2018). A Short Note about Kinetics-600. arXiv.

[44] Bhattacharyya A., Fritz M., Schiele B. (2018). Long-Term On-Board Prediction of People in Traffic Scenes under Uncertainty. arXiv.

[45] Tran D., Bourdev L., Fergus R., Torresani L., Paluri M. Learning Spatiotemporal Features with 3D Convolutional Networks. Proceedings of the 2015 IEEE International Conference on Computer Vision (ICCV).

[46] Pedregosa F., Varoquaux G., Gramfort A., Michel V., Thirion B., Grisel O., Blondel M., Prettenhofer P., Weiss R., Dubourg V. (2011). Scikit-learn: Machine Learning in Python. J. Mach. Learn. Res..

[47] Dosovitskiy A., Ros G., Codevilla F., Lopez A., Koltun V. CARLA: An Open Urban Driving Simulator. Proceedings of the 1st Annual Conference on Robot Learning (CoRL).

